# The evolution of the gliotoxin biosynthetic gene cluster in *Penicillium* fungi

**DOI:** 10.1093/g3journal/jkae063

**Published:** 2024-03-20

**Authors:** Charu Balamurugan, Jacob L Steenwyk, Gustavo H Goldman, Antonis Rokas

**Affiliations:** Department of Biological Sciences, Vanderbilt University, VU Station B #35-1634, Nashville, TN 37235, USA; Vanderbilt Evolutionary Studies Initiative, Vanderbilt University, Nashville, TN 37235, USA; Department of Biological Sciences, Vanderbilt University, VU Station B #35-1634, Nashville, TN 37235, USA; Vanderbilt Evolutionary Studies Initiative, Vanderbilt University, Nashville, TN 37235, USA; Howards Hughes Medical Institute and the Department of Molecular and Cell Biology, University of California, Berkeley, CA 94720, USA; Faculdade de Ciencias Farmacêuticas de Ribeirão Preto, Universidade de São Paulo, Ribeirão Preto, São Paulo CEP 14040-903, Brazil; Department of Biological Sciences, Vanderbilt University, VU Station B #35-1634, Nashville, TN 37235, USA; Vanderbilt Evolutionary Studies Initiative, Vanderbilt University, Nashville, TN 37235, USA

**Keywords:** comparative genomics, evolutionary biology, secondary metabolic gene clusters, duplication and loss, plant pathogen, secondary metabolism, specialized metabolism

## Abstract

Fungi biosynthesize diverse secondary metabolites, small organic bioactive molecules with key roles in fungal ecology. Fungal secondary metabolites are often encoded by physically clustered genes known as biosynthetic gene clusters (BGCs). Fungi in the genus *Penicillium* produce a cadre of secondary metabolites, some of which are useful (e.g. the antibiotic penicillin and the cholesterol-lowering drug mevastatin) and others harmful (e.g. the mycotoxin patulin and the immunosuppressant gliotoxin) to human affairs. Fungal genomes often also encode resistance genes that confer protection against toxic secondary metabolites. Some *Penicillium* species, such as *Penicillium decumbens*, are known to produce gliotoxin, a secondary metabolite with known immunosuppressant activity. To investigate the evolutionary conservation of homologs of the gliotoxin BGC and of genes involved in gliotoxin resistance in *Penicillium*, we analyzed 35 *Penicillium* genomes from 23 species. Homologous, lesser fragmented gliotoxin BGCs were found in 12 genomes, mostly fragmented remnants of the gliotoxin BGC were found in 21 genomes, whereas the remaining 2 *Penicillium* genomes lacked the gliotoxin BGC altogether. In contrast, broad conservation of homologs of resistance genes that reside outside the BGC across *Penicillium* genomes was observed. Evolutionary rate analysis revealed that BGCs with higher numbers of genes evolve slower than BGCs with few genes, suggestive of constraint and potential functional significance or more recent decay. Gene tree–species tree reconciliation analyses suggested that the history of homologs in the gliotoxin BGC across the genus *Penicillium* likely involved multiple duplications, losses, and horizontal gene transfers. Our analyses suggest that genes encoded in BGCs can have complex evolutionary histories and be retained in genomes long after the loss of secondary metabolite biosynthesis.

## Introduction

Gliotoxin is a secondary metabolite produced by certain fungi, including the major opportunistic human pathogen *Aspergillus fumigatus* (Gardiner and Howlett 2005; Raffa and Keller 2019). Secondary metabolites are bioactive molecules of low molecular weight that are not required for organismal growth but aid survival in harsh environments (Raffa and Keller 2019). Genes that participate in the biosynthesis of secondary metabolites, including gliotoxin, typically reside next to each other in fungal genomes and form biosynthetic gene clusters (BGCs) (Brown et al. 1996; Rokas et al. 2020).

Gliotoxin is a epidithiodioxopiperazine (ETP)-type fungal secondary metabolite that, in *A. fumigatus*, is biosynthesized by a 13-gene BGC (Scharf et al. 2012). Production of gliotoxin is implicated in *A. fumigatus* pathogenicity because gliotoxin suppresses the immune response of the mammalian host through diverse mechanisms, including by inhibiting protein complexes necessary for the generation of antimicrobial reactive oxygen species, decreasing cytotoxic activities of T lymphocytes, and preventing integrin activation (Yamada et al. 2000; Dolan et al. 2015; Schlam et al. 2016; Raffa and Keller 2019). The role of gliotoxin in modulating host biology suggests that it is a virulence factor and, potentially, 1 major component, or “card”, of virulence in a larger “hand” that fungi possess (Casadevall 2007; Raffa and Keller 2019). For example, virulence is attenuated in certain animal models of disease when *gliP*, the nonribosomal peptide synthetase gene involved in gliotoxin biosynthesis, is deleted (Cramer et al. 2006; Kupfahl et al. 2006; Sugui et al. 2007).

Fungi that produce gliotoxin require resistance to the toxin. Genes contributing to resistance include *gliT*, which encodes a thioredoxin reductase located within the gliotoxin BGC (Schrettl et al. 2010). Deletion of *gliT* in *A. fumigatus* results in hypersensitivity and lower resistance to the toxin (Owens et al. 2015). Other resistance genes include transcription factors, transporters, and oxidoreductases, all of which reside outside the BGC and—like *gliT*—are found in both gliotoxin-producing and nonproducing species (Castro et al. 2022). For example, the transcription factor RglT, which is the primary regulator of *gliT* (Ries et al. 2020), occurs both in *Aspergillus* species known to biosynthesize gliotoxin, such as *A. fumigatus*, as well as in species that do not biosynthesize gliotoxin, such as *Aspergillus nidulans* (Castro et al. 2022). Seven other genes are known to be regulated by *rglT* and contribute to gliotoxin resistance: *gtmA* (encodes a *bis*-thiomethyltransferase, AFUA_2G11120), *kojR* (transcription factor, AFUA_5G06800), *abcC1* (ABC-transporter, AN7879/AFUA_1G10390), *mtrA* (methyltransferase, AN3717/AFUA_6G12780), AN9051 (oxidoreductase, AFUA_7G00700), AN1472 (MFS transporter, AFUA_8G04630), and AN9531 (NmrA-like family transcription factor, AFUA_7G06920) (Castro et al. 2022).

Previous research on *Aspergillus* showed that gliotoxin resistance genes are more widely conserved than gliotoxin BGC genes; for example, non-gliotoxin-producing species typically lack the entire BGC but harbor gliotoxin resistance genes (Ries et al. 2020; Steenwyk et al. 2020b; Castro et al. 2022). Some *Penicillium* species, such as *P. decumbens*, can produce gliotoxin (Feng et al. 2018), but the distribution and evolution of gliotoxin BGCs in other filamentous fungal lineages, such as *Penicillium*, remains unknown. To address this question, this study examined the evolutionary conservation of homologs of the gliotoxin BGC and resistance genes in *Penicillium*, a genus of fungi that is closely related to *Aspergillus.*

Examination of the evolutionary conservation of homologs of the gliotoxin BGC and gliotoxin resistance genes among 35 strains of 23 *Penicillium* species revealed that most *Penicillium* genomes encode fragmented gliotoxin BGCs; 2 lacked gliotoxin BGCs altogether. In contrast, some *Penicillium expansum* strains encoded 2 homologous gliotoxin BGCs. Codon optimization analysis revealed that genes *in Penicillium* BGCs are lowly optimized, consistent with the observed BGC fragmentation. In contrast, gliotoxin resistance genes are codon-optimized, suggesting that *Penicillium* species encounter exogenous gliotoxin in their environments. Examination of evolutionary rates revealed that genes from highly fragmented gliotoxin BGCs evolved at significantly higher rates than genes from lesser fragmented BGCs, suggesting that more fragmented BGCs experienced relaxation of selective constraints for longer. Gene tree–species tree reconciliation analyses inferred that the evolution of homologs of genes in the gliotoxin BGC of the genus *Penicillium* was complex, involving gene duplications, losses, and horizontal transfers, even though the ability of these fungi for gliotoxin resistance presumably remained conserved.

## Materials and methods

### Data collection and quality assessment

The genomes and gene annotations of 35 *Penicillium* strains from 23 species as well as of 2 outgroups (*A. fumigatus* and *Aspergillus fischeri*) were retrieved from NCBI (https://www.ncbi.nlm.nih.gov/) (Supplementary Table 1).

Genome assembly and annotation quality were examined to evaluate whether the dataset is sufficient for comparative genomics. The quality and characteristics of the genomes (N50, L50, assembly size, number of scaffolds, and gene count) were evaluated using BioKIT (v0.1.0) (Steenwyk et al. 2022) (Supplementary Fig. 1). The average N50 value was ∼1.85 Mb bases, where 46% of genomes consisted of N50 values greater than 1 Mb, and the lowest N50 value was 31,119 bases for *P. expansum* CMP 1. Gene annotation completeness was assessed using BUSCO (v5.0.0) (Waterhouse et al. 2018) (Supplementary Fig. 2). BUSCO uses a predetermined set of near-universally conserved single-copy genes (or BUSCO genes) to identify their presence in a query proteome (characterized as single-copy, duplicated, or fragmented) or absence. The 4,181 BUSCO genes from the Eurotiales OrthoDB dataset were used (Manni et al. 2021; Zdobnov et al. 2021). Nearly all the genomes have high BUSCO gene coverage (average: 95.9% ± 3.1%), with the lowest percentages being for *Penicillium coprophilum* (87.9%) and *P. decumbens* (85.3%).

### Identification and characterization of gliotoxin BGC and resistance homologs

#### Identification of gliotoxin BGC and resistance homologs

The representative gliotoxin BGC (BGC0000361, download date: April 2022) from the *Aspergillus fumigatus* Af293 reference strain was downloaded from the Minimum Information about a Biosynthetic Gene Cluster (MiBIG) database (Kautsar et al. 2020). Command-line NCBI BLASTP (Camacho et al. 2009) searches for the Af293 gliotoxin BGC against the proteome of each species were executed. Highly similar sequences were identified using an expectation value threshold of 1e^−4^ and a query coverage of 50%. The resulting BLAST outputs were then cross-referenced with the NCBI feature table file, which contains genome location information for each gene, and parsed to identify clusters of homologs. Lesser fragmented BGCs are defined as having at least 7/13 genes from the query gliotoxin BGC present, including *gliP*, encoding the core nonribosomal peptide synthetase (Castro et al. 2022); more fragmented clusters are defined as having between 3 and 6/13 genes from the gliotoxin BGC without a requirement for this cluster to include *gliP*. When identifying BGCs, up to 4 genes between each pair of adjacent homologs were allowed using the *A. fumigatus* Af293 BGC from the MiBIG database (Kautsar et al. 2020) as reference (Castro et al. 2022).

To rule out gene annotation errors in cases where genes were inferred to be absent, command-line NCBI tBLASTn searches for the Af293 gliotoxin BGC against the genome sequences were conducted. Highly similar sequences were identified using an expectation value threshold of 1e^−10^. The resulting outputs were analyzed, and no new presence/absence information was found.

Sequence similarity searches were also conducted for homologs of 8 gliotoxin resistance genes (*abcC1*/AFUA_1G10390, *mtrA*/AFUA_6G12780, AFUA_7G00700, AFUA_8G04630, AFUA_7G06920, *rglT*/AFUA_1G09190, *gtmA*/AFU2G11120, *kojR*/AFUA_5G06800), 3 of which were transcription factors (AFUA_7G06920, *rglT*, *kojR*). The gene query sequences for all 8 of these gliotoxin resistance genes were retrieved from the *A. fumigatus* Af293 genome (Nierman et al. 2005). An expectation value threshold of 1e^−3^ and a query coverage threshold of 50% were used; a lower query coverage threshold of 40% was used for the 3 transcription factors.

#### Estimating codon optimization

To estimate the potential functional significance of the partial gliotoxin BGCs present in *Penicillium* genomes, mean gene-wise relative synonymous codon usage (gRSCU) was determined for each clustered *gli* homolog across all proteomes using BioKIT (Steenwyk et al. 2022). This provides insight into how codon usage bias influences the expression level of a particular homolog. The percentile rankings of each of the present and clustered *gli* homologs were calculated using the R package *dplyr* (v1.0.9) (Wickham et al. 2022), and these values, for each species, were then plotted using the R package *ggplot2* (Wickham 2016).

#### Synteny analysis

Alignments of representative *Penicillium* genomes with lesser and more fragmented gliotoxin BGCs were generated using a GenomeDiagram in Biopython (Cock et al. 2009). Five genomes (*A. fumigatus* Af293, *Penicillium flavigenum* IBT 14082, *Penicillium roqueforti* FM164, *Penicillium nordicum* DAOMC 185683, and *P. expansum* CMP1) with the largest number of different, homologous *gli* cluster genes above 7, and including *gliP*, were chosen to visualize the conservation of synteny of lesser fragmented gliotoxin BGCs across the phylogeny. Similarly, the 5 genomes (*Penicillium steckii* IBT 24891, *Penicillium vulpinum* IBT 29486, *Penicillium rubens* 43M1, *Penicillium camemberti* FM 013, and *Penicillium italicum* PHI 1) with the greatest number of different, homologous *gli* cluster genes above 3 and below 7, and not needing to include *gliP*, were chosen to visualize synteny of mostly fragmented BGCs across the phylogeny.

### Phylogenetic analysis

#### Species tree inference

The evolutionary relationships of *Penicillium* species were obtained from a previous study (Steenwyk et al. 2019) using treehouse (Steenwyk and Rokas 2019). For 3 species with population-level data, within-species relationships were inferred using phylogenomics. To do so, protein sequences of BUSCO genes were first aligned using MAFFT (v7.490) with the *–auto* parameter (Katoh and Standley 2013). Codon-based alignments were generated by threading the corresponding DNA sequences onto the protein alignment with the *thread_dna* function in PhyKIT (v1.11.2) (Steenwyk et al. 2021). The resulting nucleotide alignments were trimmed using ClipKIT (v1.3.0) (Steenwyk et al. 2020a) with default parameters. The resulting aligned and trimmed sequences were concatenated into a supermatrix with 8,124,861 sites using the *create_concat* function in PhyKIT. The concatenated matrix was then analyzed with IQ-TREE 2 (v2.0.6), a software that implements a maximum likelihood framework for inferring phylogenies. All other evolutionary relationships between species were constrained following the relationships inferred in a previously published study (Steenwyk and Rokas 2019). The best-fitting substitution model (GTR + F + I + G4) was determined using ModelFinder (Kalyaanamoorthy et al. 2017).

#### Single-gene tree inference

To infer the evolutionary history of *gli* homologs, the translated amino acid sequences of individual *gli* genes (both clustered and unclustered) were compiled and aligned with MAFFT (v7.490) using the *–auto* parameter (Katoh and Standley 2013). The corresponding nucleotide sequences for each file were obtained from the CDS files for each species, using the *faidx* function of BioKIT (v0.1.0) (Steenwyk et al. 2022). These nucleotide sequences were then threaded onto the protein alignments using the *thread_dna* function of PhyKIT (Steenwyk et al. 2021), resulting in a codon-based alignment. All individual codon-based gene alignments were trimmed with ClipKIT (Steenwyk et al. 2020a) with default parameters. The trimmed alignments were used to construct a phylogeny using IQ-TREE 2 (Minh et al. 2020). The best-fitting substitution model was chosen for each *gli* gene using Bayesian information criteria (BIC) implemented in ModelFinder (Kalyaanamoorthy et al. 2017) from IQ-TREE 2. Branch support in each phylogenetic tree was assessed by 1,000 bootstraps using ultrafast bootstrapping approximation (Hoang et al. 2018). Tree visualization was carried out using the R packages *ape* (v5.6.2) (Paradis and Schliep 2019) and *phytools* (v1.0.3) (Revell 2012).

To characterize variation in the evolution of individual homologous genes of the gliotoxin BGC, the trimmed alignments and maximum-likelihood trees from IQ-TREE 2 were used as input into the *evolutionary_rate*, *total_tree_length*, and *pairwise_identity* functions of PhyKIT to estimate 2 tree-based measures of evolutionary rate and 1 sequence-based measure. Evolutionary rate is defined as the total tree length divided by the number of terminals (Telford et al. 2014; Steenwyk et al. 2021). The total tree length is the sum of all branches (Steenwyk et al. 2021).

#### Tree topology testing

Diverse evolutionary scenarios can result in single genes that have evolutionary histories distinct from organismal history (Steenwyk et al. 2023). To shed light on the evolutionary history of the duplicated *P. expansum* BGCs, topology testing was conducted. Specifically, we tested if duplication occurred within the lineage of *P. expansum* or deeper in the tree before the diversification of *P. expansum* isolates. To do so, IQ-TREE 2 (Minh et al. 2020) was used to compute log-likelihoods of a constrained tree (monophyly of homologs) and the observed tree in which a polyphyly of homologs in both clusters is seen (inconsistent with the known species tree). For each comparison, 1,000 RELL replicates (Kishino et al. 1990) were performed. The AU test results (Shimodaira 2002) was used for comparison.

#### Gene tree–species tree reconciliation under gene duplication, transfer, and loss

Considering the observed variation in *gli* gene tree topologies relative to the species phylogeny (Steenwyk et al. 2019), we used GeneRax, a tool for species tree-aware maximum likelihood-based gene family tree inference under gene duplication, transfer and loss (Morel et al. 2020). Two models were employed to reconcile the gene family trees with the species tree; the Undated DTL model that accounts for duplications, losses, and transfers, and the UndatedDL model that accounts for duplications and losses only. Both were run using the SPR tree search algorithm. The main outputs from this analysis are the inferred gene trees reconciled with the species tree, in RecPhyloxml format, along with reconciliation likelihood scores (1 per model). We then visualized these trees via Thirdkind, a program that builds svg representations of gene trees that are reconciled with the species phylogeny through the inference of loss, duplication, and transfer events (Penel et al. 2022).

## Results and discussion

### The gliotoxin BGC is fragmented in *Penicillium* species

**Fig. 1.**
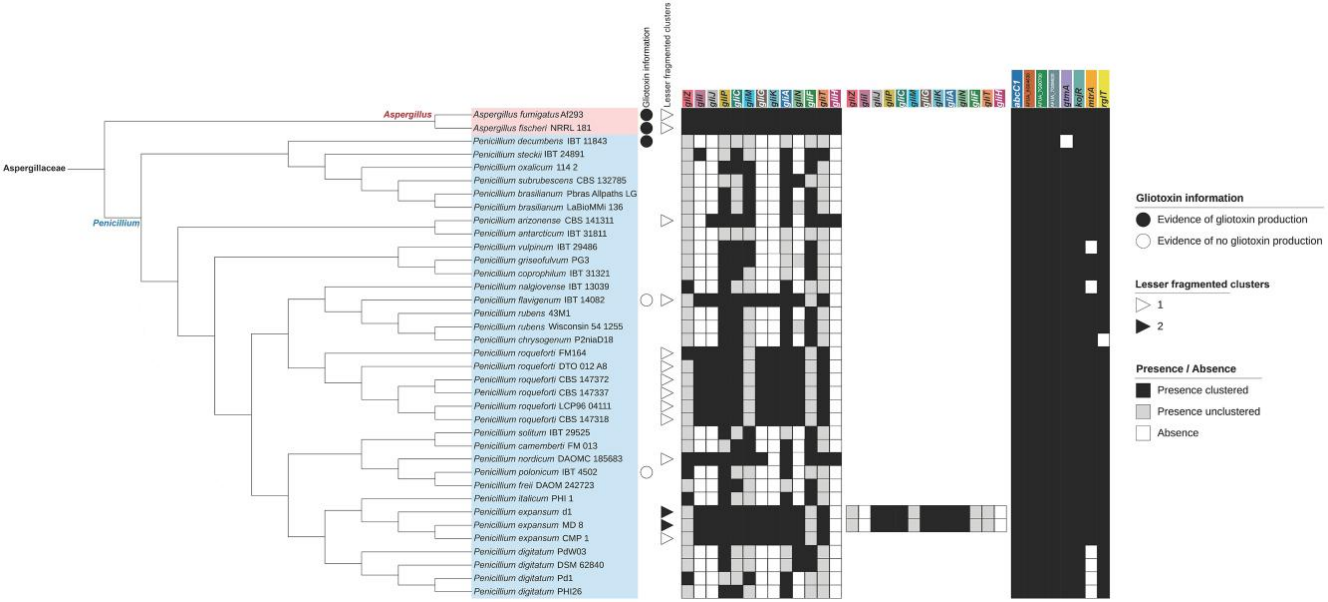
Phylogeny of *Penicillium* genomes. Different genera are depicted using different-colored boxes. The clades of the genera *Aspergillus* and *Penicillium* are indicated on the phylogeny. Shaded circles next to species/strain names indicate gliotoxin production information from the literature, or lack thereof (Fischer et al. 2000; Spikes et al. 2008; Knowles et al. 2020; Redrado et al. 2022). Shaded triangles in the second column depict number of clusters identified. Remaining color strips depict gene presence clustered (black), presence unclustered (gray), and absence (white) according to the requirements outlined in the *Materials and methods* section. Analyses of the evolutionary history of gli homologs using species tree-aware maximum likelihood-based gene family tree inference suggest that they have a complex evolutionary history characterized by gene duplications, gene losses, and horizontal transfers (Supplementary Figs. 24–36).

Presence/absence data of homologs of the 13 genes in the gliotoxin BGC among the 23 *Penicillium* species analyzed reveals that the cluster is largely fragmented in the genus *Penicillium* (Fig. 1). The genomes of 12 strains from 5 *Penicillium* species (P*enicillium arizonense*, *P. flavigenum*, *P. roqueforti*, *P. nordicum*, *P. expansum*), encoded lesser fragmented BGCs; the genomes of the other 23 strains spanning 18 *Penicillium* species encoded more fragmented BGCs (Supplementary Figs. 3–15). Two lesser fragmented BGCs, which contained 10/13 genes and 7/13 genes, were identified in *P. expansum* strains d1 and MD 8, respectively. Regardless of the number of lesser fragmented BGCs found, to our knowledge, none of the *Penicillium* species in question are known to produce gliotoxin, except *P. decumbens* (Feng et al. 2018), suggesting that the absence of clustering in this species may be due to strain heterogeneity and requires further exploration.

### Gliotoxin resistance genes are broadly conserved

The presence/absence results of homologs of the 8 gliotoxin resistance genes showed that all species possessed *abcC1*, *AFUA_8G04630*, *AFUA_7G00700*, *AFUA_7G06920*, and *kojR* homologs (Fig. 1, Supplementary Figs. 16–23). In addition, only *Penicillium* species with mostly fragmented gliotoxin BGCs lacked at least 1 resistance gene, such as *gtmA*, *mtrA*, and *rglT*. *Penicillium chrysogenum* lacked both *rglT* and *gliT*, an observation consistent with the transcriptional dependency of *gliT* to *rglT* (Ries et al. 2020). These results raise the hypothesis that the genetic mechanisms likely involved in gliotoxin resistance have been largely maintained throughout *Penicillium* evolution, whereas BGCs are more readily lost.

### *Gli homologs* in *Penicillium* species had a complex history involving gene duplications, losses, and horizontal transfers

Duplication, loss, and horizontal gene transfer of fungal BGCs and their genes are well established (Wisecaver and Rokas 2015; Rokas et al. 2020). To examine whether *gli* homologs had more complex evolutionary histories involving duplication and horizontal transfer, we reconciled the history of each gene with the species history using 2 models in the GeneRax software: a model that reconciles *gli* gene histories to the species phylogeny by allowing duplications and losses (Undated DL model) and a model that allows duplications, transfers, and losses (Undated DTL model). The DTL model had a significantly better fit than the DL model (log likelihood score of −1837.01 vs −2437.53, respectively), suggesting that the history of *gli* homologs included gene duplications, losses, and horizontal gene transfers (Supplementary Figs. 24–36).

### *Penicillium* species experienced changes in gliotoxin BGC synteny over time

**Fig. 2.**
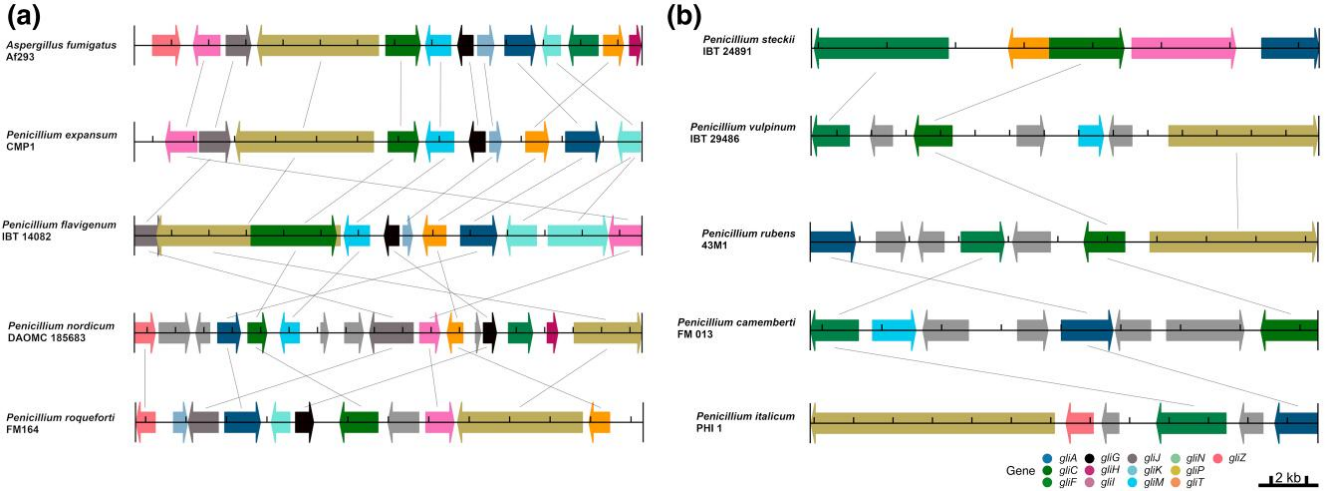
Conservation of gliotoxin BGC synteny for representative *Penicillium* species. Synteny analysis of representative genomes with lesser fragmented a) and more fragmented b) gliotoxin BGCs. Each interval along the track represents 2 kb.

Examination of the synteny of genes in the BGC showed that it is mostly conserved and similar to the arrangement of the *A. fumigatus* Af293 gliotoxin BGC across representative, lesser fragmented BGCs, such as *P. flavigenum* IBT 14082 *and P. expansum* CMP 1 (Fig. 2). In contrast, there is extensive divergence in synteny conservation among more fragmented BGCs (Fig. 2). More specifically, 12 out of 35 *Penicillium* species/strains were found to have a lesser fragmented, homologous BGC. Two strains of *P. expansum* (d1 and MD 8) were found to have 2 BGCs, indicating variation exists in gene presence/absence within the species. For example, *P. roqueforti* shows population variation in the presence of *gliZ*, a major transcriptional regulator of gliotoxin biosynthesis (Bok et al. 2006); 5 of the 6 strains of *P. roqueforti* examined lack *gliZ*. Thus, we hypothesize that the ancestor of *P. roqueforti* had a *gliZ* homolog, but the gene was lost over time in most of the strains, highlighting the importance of population-level sampling. Overall, it can be seen that the gliotoxin BGC has experienced relocations, duplications, and horizontal transfers of its genes, specifically in *P. expansum* strains d1 and MD 8, a finding consistent with observations from many other secondary metabolite-producing BGCs (Rokas et al. 2018).

### Contrasting codon optimization in *gli* genes vs gliotoxin resistance genes

**Fig. 3.**
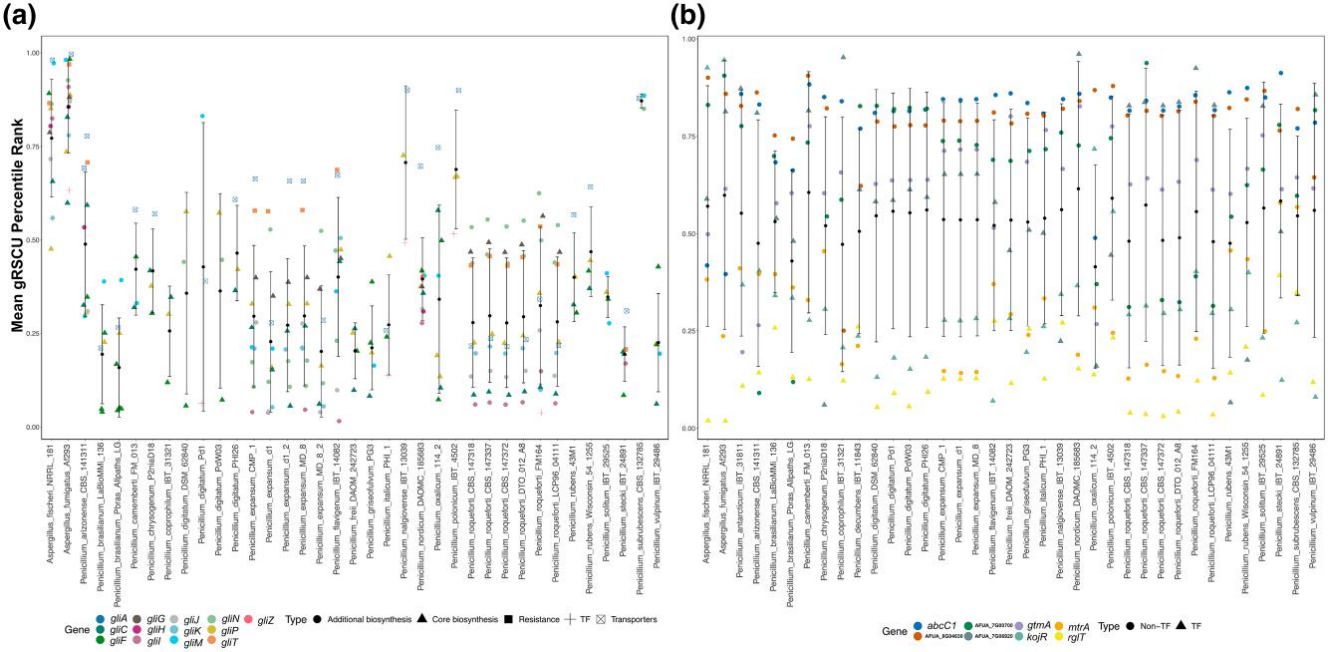
Gene-wise relative synonymous codon usage (gRSCU) for gliotoxin BGC and resistance genes. a) Percentile rankings of gene-wise relative synonymous codon usage (gRSCU) among gliotoxin BGC genes, in comparison to all other genes. Types/functionality of each gene of the gliotoxin BGC is depicted by shape in the categories of “Core biosynthesis”, “Additional biosynthesis”, “Resistance”, “Transcription Factor”, and “Transporter”. b) Percentile ranking of gene-wise relative synonymous codon usage (gRSCU) among gliotoxin resistance genes, in comparison to all other genes. Types/functionality of each resistance gene is depicted by shape in the categories of “Non-Transcription Factor and Transcription Factor”.

Compared to the 2 outgroup *Aspergillus* species—*A. fumigatus* and *A. fischeri*—gliotoxin BGC genes found in *Penicillium* species have much lower gRSCU percentile values, a measure of codon optimization (Steenwyk et al. 2022) (Fig. 3). Specifically, the mean gRSCU percentile rank of gliotoxin BGC genes among the *Aspergillus* outgroups is 0.81, while that among the *Penicillium* species is 0.35; these scores suggest that *gli g*enes from *Aspergillus* are more codon-optimized than *gli* genes from *Penicillium*. Regardless of mean gRSCU values, *gliT* and *gliA* homologs, when present, are ranked consistently with the top 3 to 4 clustered genes. However, when considering resistance genes, the spread and range of their gRSCU values are similar across all species. The mean gRSCU percentile rank of homologs of gliotoxin resistance genes among the *Aspergillus* outgroups is 0.58, while that among the *Penicillium* species is 0.53. The similar percentile ranking of transporter and resistance genes between *Penicillium* species and *Aspergillus* species known to be resistant to exogenous gliotoxin suggests that these *Penicillium* species may ecologically encounter exogenous gliotoxin. This finding further supports the hypothesis that gliotoxin resistance has been maintained throughout *Penicillium* evolution, whereas BGC production has not.

### Homologs of *gli* genes in lesser fragmented clusters are evolving at a slower rate than more fragmented clusters

In the comparison of tree-based and sequence-based measures of evolutionary rate, homologs of *gli* genes from lesser fragmented clusters are evolving at a significantly slower pace (*P* < 0.0001; two-way ANOVA) than those from more fragmented clusters across all 3 metrics (Fig. 4, Supplementary Figs. 3–15).

**Fig. 4.**
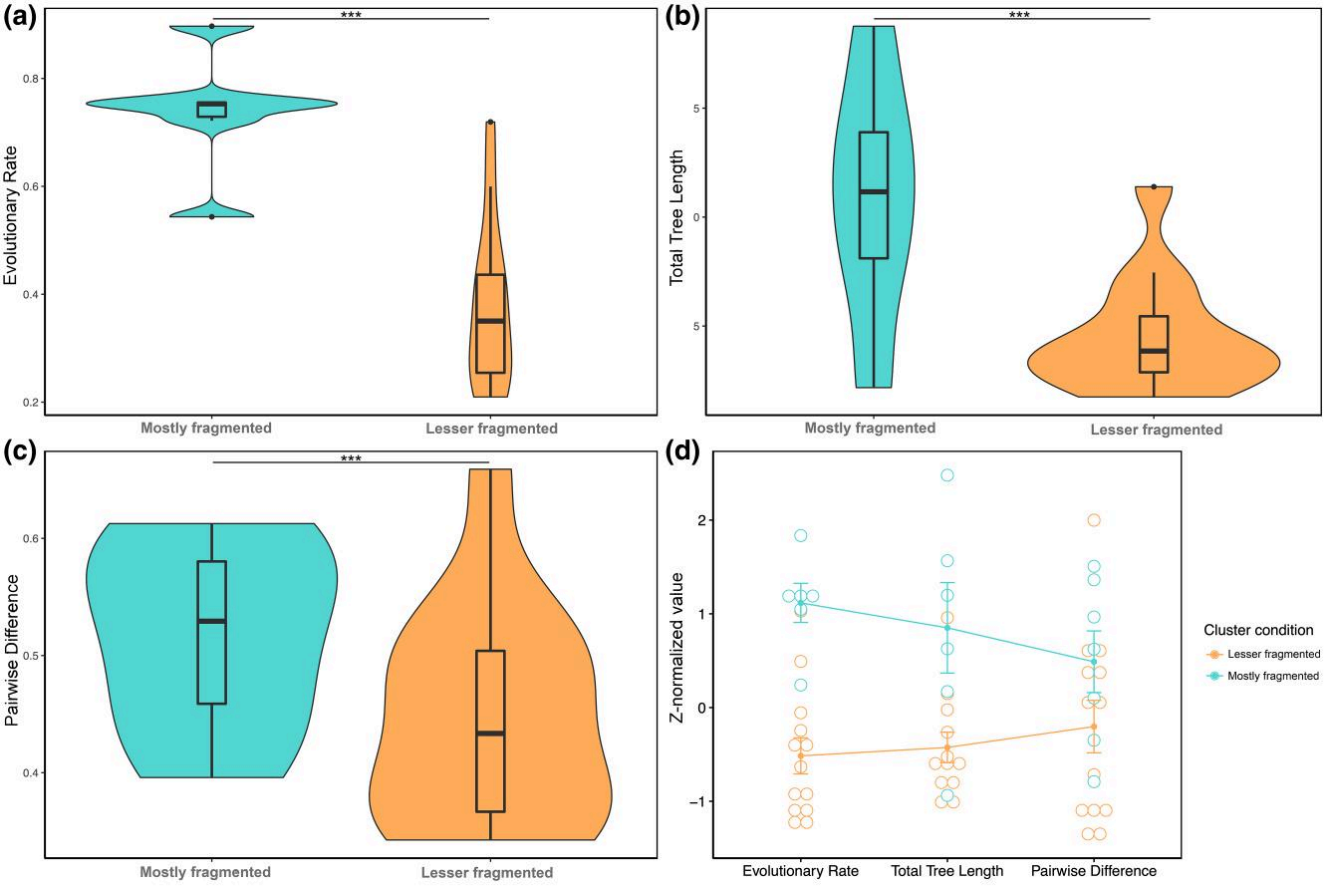
Evolutionary rate comparison across gliotoxin BGCs. Multi-method comparison of evolutionary rates between lesser fragmented and mostly fragmented gliotoxin BGCs. Lesser fragmented clusters were required to contain a gliP homolog and at least 7 different genes of the cluster. Mostly fragmented clusters had no requirement to contain a gliP homolog and only needed to contain at least 3 different genes of the cluster. a) Comparison of evolutionary rates, as a function of total tree length divided by the number of taxa, between lesser fragmented and mostly fragmented gliotoxin BGCs. b) Comparison of total tree length between lesser fragmented and mostly fragmented gliotoxin BGCs. c) Comparison of pairwise identity between lesser fragmented and mostly fragmented gliotoxin BGCs. d*)* Comparison of the *Z*-normalized values of each metric between lesser fragmented and more fragmented gliotoxin BGC evolutionary rate values via a 3-way interaction plot.

### A complex evolutionary history of the *P. expansum* gliotoxin BGCs

A tree topology test was conducted to illuminate the evolutionary origin of the multiple gliotoxin BGCs encoded in *P. expansum* (Supplementary Fig. 37). The maximum likelihood phylogeny suggests that a horizontal gene transfer or a combination of duplication and loss occurred between *P. flavigenum* and *P. expansum*. A combination of duplication and loss would be supported by monophyly of *P. expansum gli* gene homologs. After conducting tree topology tests comparing log likelihood values between the maximum likelihood phylogeny and an alternative tree wherein *P. expansum* homologs were constrained to be monophyletic, the likelihood scores of the unconstrained and constrained topologies were not significantly different, which suggests that the hypothesis that a combination of duplication and loss occurred within the *P. expansum* lineage cannot be rejected (*P* > 0.05, Approximately Unbiased test, Supplementary Table 2). In contrast, the GeneRax analyses (Supplementary Figs. 24–36) (Morel et al. 2020; Penel et al. 2022), support a scenario where genes present within the multiple gliotoxin BGCs in *P. expansum* were transferred largely between *P. flavigenum* and *P. expansum* d1, and between *P. expansum* d1 and *P. expansum* MD8 (e.g. Supplementary Fig. 31).

## Conclusions

The evolution of *gli* homologs across the genus *Penicillium* likely involved multiple duplications, losses, and horizontal gene transfers. The presence/absence results of homologs of the 8 resistance genes suggest that their origins predate the *Aspergillus* and *Penicillium* genera suggesting that resistance has long been important among these species. The genes in *Penicillium* gliotoxin BGCs are less codon-optimized (gRSCU percentile rank mean: 0.35) compared to their *Aspergillus* counterparts (gRSCU percentile rank mean: 0.81) suggesting that *gli* genes may be more important for *Aspergillus* ecology. In contrast, resistance genes are similarly codon-optimized, suggesting that resistance is relevant to the lifestyle of both genera.

Although informative, this work only utilizes publicly available protein annotations of biotechnologically and medically relevant *Penicillium* fungi, making it important to expand upon the species/strains studied. Moreover, this same targeted gliotoxin analysis within a larger phylogeny of *Aspergillus* species, for which there is greater evidence of the production of this secondary metabolite, may be helpful. An analysis of *gli* homologs across the fungal kingdom, including careful delineation of *gli* orthologs and paralogs, would also provide us with more insight into the evolutionary mechanisms that gave rise to the gliotoxin BGC and to the diversity of *gli* homologs. In addition, expanding on the causes of conservation of lesser fragmented gliotoxin BGCs within a variety of *Penicillium* strains may be important, especially because evidence of production is lacking. However, it is important to note that, although there is no evidence of gliotoxin production for many of these species, this does not necessarily mean that they cannot produce the toxin (e.g. they may biosynthesize gliotoxin only under certain, yet to be identified, conditions).

## Data Availability

All data necessary for confirming the article's conclusions are present within the article, figures, tables, and supplemental material (found at this Figshare repository: 10.6084/m9.figshare.23600772).
